# A Lesson to Learn from the History of American Physicians

**DOI:** 10.31729/jnma.5097

**Published:** 2020-07-31

**Authors:** Manoj Khadka

**Affiliations:** 1 Nepalese Army Institute of Health Sciences, Sanobharyang, Kathmandu, Nepal

As per the American Medical Association Physicians' Professional Data (AMA-PPD) file, the number of international medical graduates (IMGs) in the United States were 215,576 in 2004,which is about 2.4 times its 1978 size.^1^ So, the United States seems to be a dream country for many medical students all over the world.

Many medical students in Nepal too are attracted to the United States. In a retrospective cohort study done among 710 graduate doctors of the Institute of Medicine from 1983-2004, 256 (36.1%) were working in foreign countries, out of which 188 (73%) were in the United States.^2^

But why is that many medical students worldwide are interested to work in the United States? In a study conducted at Dow Medical College of Dow University of Health Sciences, Pakistan in 2012, 60.4 % of the students wanted to work abroad and the United States was the country of choice for the majority. Four most common reasons given by students who wished to migrate were high paid salary abroad, quality of training, job satisfaction, and a stronger way of life.^3^ So, there could be various pull (conditions in the target country) as well as push (conditions in the country of origin) factors for the physicians' migration.

The increased interest of medical students to work in the United States leads to a question, Was the United States like today in past centuries too regarding the field of medicine? To quench this thirst of curiosity, let's turn the pages in the history of medicine of the USA around two centuries back.

There was a time in the USA when admission to the medical school required the applicant's ability to pay the fees. Anatomy was taught without dissection. The percussion, which is commonly done nowadays in physical examination of the patient, was hardly performed by physicians. The medical instruments like the thermometer, stethoscopes were rarely used.^4^ Can you even believe it!

## CIVIL WAR: THE MIDDLE AGES OF MEDICINE IN THE UNITED STATES

During the Civil war in the USA (1861-1865), it was estimated that 50,000 Confederate soldiers died from wounds and 150,000 from the disease while 110,000 Union soldiers died from wounds and 225,000 from the disease.^4^ Around twice as many soldiers died of disease than from the battle itself. Some contributing factors to deaths were poor sanitation, overcrowded camps, poor diet, wound infections, and limited specific treatments.^5^ Though there were some medical and surgical advances during the Civil War, it pointed up the weakness of American physicians and surgeons.

Isn't it hard to believe that it's a history of America? You might be wondering how a drastic change was made in the field of medicine in the USA. Well, if that's your curiosity then here is the answer for you. Enjoy reading!

## INFLUENCE OF THE FRENCH SCHOOL

From 1800 to 1850, physicians from not only America but the whole world visited Paris for their further studies. Some renowned clinicians of Paris were Corvisart (Napoleon Bonaparte's physician who popularized percussion in 1808 that was discovered by Auenbrugger in 1761), Laennec (discovered stethoscope in 1816), surgeon Dupuytren. French's strength was the observation, be it clinical observation or the autopsy. This influenced American physicians to establish the Society for Medical Observation in 1832 in America. The American physicians also doubted experimentation probably due to the influence of the French School as French didn't trust the experimental laboratory investigation.^4^

## INFLUENCE OF THE GERMAN SCHOOL

As the French depended upon observation for diagnosis and didn't investigate the cause of disease, the focus of physicians around the world shifted to Germany from Paris starting in the 1850s. Germans used experimental laboratory investigations and focused on medical research to solve the puzzles of medicine. This influenced American physicians who went to Germany for their postgraduate education to establish the American Society for Clinical Investigation in 1908. They also played a key role to bring a significant change in American medical education at Harvard, Pennsylvania, Michigan, and Johns Hopkins university.^4^

## THE RISE OF JOHNS HOPKINS HOSPITAL AND MEDICAL SCHOOL

The changes that began at the University of Harvard, Pennsylvania, and Michigan led to the opening of the Johns Hopkins Medical School in 1893 (four years after the establishment of Johns Hopkins hospital in 1889). The four founding physicians of Johns Hopkins, popular as “Big Four” were pathologist William Henry Welch, internist William Osler, surgeon William Stewart Halsted, and gynecologist Howard Kelly.^6^

When I first heard about John Hopkins, I thought that he might be a great physician of his times and that could be the reason to name a hospital and university after his name as a tribute. But I was astonished when I knew that Johns Hopkins was a powerful financier and philanthropist in Baltimore. As he couldn't receive the education he wished for and also witnessed the epidemics in Baltimore, he wanted to provide a university and a hospital for others. So, he had arranged for $7 million to be split evenly between Johns Hopkins University and the Johns Hopkins Hospital.^7^ He also specified that a school of medicine linked with a university, accompany the hospital. This innovative idea later became the model for all academic institutions.^8^

Johns Hopkins medical school had strict admission requirements, two years of basic science training, and two years of clinical experience at the hospital bedside. The research was given high priorities. Medical students rather than a passive observer became active participants, taking histories, and doing physical examinations.^4^ These things which were practiced a long time back, became the foundation for modern medicine today.

## WAY FORWARD

Isn't the history of medicine in the USA really interesting? Having read this long history from the French School and the German School to the establishment of John Hopkins medical school in 1893, it's clear that studying abroad is a good idea as it helps to build up our medical knowledge and sharpen clinical skills. But it's best if we apply the same in our motherland and help improve the medical field of our home country like the American physicians did after returning from France and Germany.

It's high time to bring changes in medical education in Nepal and make students able to think critically rather than just providing them bulky knowledge of facts.

Let's be the one to create the history rather than just joyfully reading it.

## Conflict of Interest

**None.**
